# EgKT2-1, an inwardly rectifying Shaker-type potassium channel in oil palm that correlates with oil yield

**DOI:** 10.1007/s00299-026-03975-7

**Published:** 2026-09-30

**Authors:** Houssein Monder, Sandra Espeout, Sabine D. Zimmermann, Norbert Billotte, Jean-Luc Verdeil, Stéphanie Bocs, Jean Ollivier, Reinout Impens, Florence Jacob, Isabelle Gaillard, Teresa Cuéllar

**Affiliations:** 1 IPSiM, University of Montpellier, CNRS, INRAE, Institut Agro, Montpellier, France; 2 CIRAD, UMR AGAP Institut, 34398 Montpellier, France; 3 UMR AGAP Institut, University Montpellier, CIRAD, INRAE, Institut Agro, Montpellier, France; 4 CIRAD, UMR ABSys, 34398 Montpellier, France; 5 UMR ABSys, University Montpellier, CIHEAM-IAMM, CIRAD, INRAE, Institut Agro, Montpellier, France; 6 SIAT Group, Presco Plc, Benin City, Nigeria; 7 PalmElit SAS, 34980 Montferrier-Sur-Lez, France

**Keywords:** *Shaker* K^+^ channel, Inward rectifier, Potassium uptake, *Elaeis guineensis*, Oil yield

## Abstract

The oil extracted from the mesocarp of oil palm (*Elaeis guineensis*, Jacq.) drupes is the world’s leading source of vegetable oil. Oil palm cultivation requires large amounts of mineral nutrients supplied through fertilization, representing a major production cost. Among essential nutrients, potassium (K⁺) plays a critical role in plant growth and fruit development. However, the mechanisms underlying K⁺ transport and its relationship with yield remain poorly understood. Here, we identified and characterized *EgKT2-1*, a Shaker-like potassium channel belonging to the AKT2-like subfamily. Quantitative PCR analyses showed that *EgKT2-1* is expressed in leaves, meristem, and during early fruit development, with higher transcript abundance during early fruit development coinciding with increased K⁺ accumulation. In situ hybridization localized *EgKT2-1* transcripts to the root cortex and phloem tissues, leaf palisade parenchyma, and mesocarp cells during early fruit development. These expression patterns are consistent with a potential contribution of *EgKT2-1* to K⁺ transport and allocation between source and sink tissues. Although sequence similarity places *EgKT2-1* within the weakly rectifying AKT2-like subfamily, electrophysiological characterization in *Xenopus laevis* oocytes revealed inwardly rectifying channel activity, suggesting a potential role in K⁺ uptake. *EgKT2-1* expression was higher in an oil palm progeny C3 which exhibited greater K⁺ allocation to aerial organs and greater oil yield. Together, these findings identify EgKT2-1 as an inwardly rectifying K⁺ channel potentially involved in K⁺ transport and redistribution during oil palm development and suggest a correlation between *EgKT2-1* expression, K⁺ allocation, and oil yield variation.

**Supplementary Information:**

The online version contains supplementary material available at https://doi.org/10.1007/s00299-026-03975-7.

## Key message

***EgKT2-1*, an inwardly rectifying** ***Shaker*** **K⁺ channel in oil palm, is expressed in roots, leaves, and early fruit development suggesting involvement in K⁺ allocation during fruit development and oil accumulation.**

**Supplementary Information:**

The online version contains supplementary material available at https://doi.org/10.1007/s00299-026-03975-7.

## Introduction

The oil palm (*Elaeis guineensis*, Jacq.) is a monocotyledonous plant whose fruits are named drupes growing in bunches. The drupe is composed of a thick fleshy mesocarp, which is exceptionally rich in oil, surrounded by the hardened endocarp (shell) that encloses the kernel and a tiny embryo. Palm oil, which is extracted from the mesocarp, is the world’s leading source of vegetable oil, accounting for approximately 34% of global vegetable oil production, and the crude oil is naturally rich in vitamin E and provitamin A (United States Department of Agriculture Economic Research Service (USDA ERS), 2026). Despite accounting for only approximately 8–9% of the global harvested area devoted to major oil crops, oil palm exhibits unmatched oil productivity, yielding around 3.3–4.0 t oil ha⁻^1^ yr⁻^1^, compared with approximately 0.5–0.6 t oil ha⁻^1^ yr⁻^1^ for soybean, 0.6–0.9 t oil ha⁻^1^ yr⁻^1^ for sunflower, and 0.8–1.0 t oil ha⁻^1^ yr⁻^1^ for rapeseed (Food and Agriculture Organization of the United Nations (FAO), 2026; Murphy 2025).

Oil palm fruits ripen around 160 days after anthesis (DAA). During early fruit development, i.e., between 30 and 60 DAA, fruit size and mass increase substantially. More generally, increases in fruit size during early development are associated with cell division and expansion, processes regulated by phytohormones including auxins, gibberellins, and cytokinins (Karim et al. 2024). In oil palm, this phase is followed by a latency period (60–100 DAA), during which hormonal activity associated with fruit growth decreases. From 120 DAA onward, ripening begins, accompanied by a strong increase in ethylene and abscisic acid (ABA) and the onset of fatty acid accumulation. Carotenes and oil accumulate during maturation from approximately 140 DAA (Tranbarger et al. 2011). To reach high yields, oil palm requires large quantities of fertilizer (Ollivier et al. 2017). Among the provided inputs, potassium (K^+^) is an essential macronutrient for oil palm, influencing vegetative growth, bunch and drupe production, and overall yield (Corley and Tinker 2015; Husri et al. 2020). As the most abundant cytosolic cation, K⁺ participates in highly integrated functions such as the control of stomatal opening and gas exchange, chlorophyll assimilation, cell turgidity, raw sap rise or phloem sap circulation, cell elongation, and plant growth. It plays a key role in the plant’s adaptation to variations in water availability in the environment. In oil palm plantations, in order to increase oil production, the average K^+^ quantity supplied is 0.52 t K^+^ fertilizer ha^−1^ year^−1^, which can account for up to 50% of the total production costs (Husri et al. 2020). Optimizing K^+^ use efficiency is thus critical to reduce fertilizer dependency and improve sustainability.

Some molecular knowledge of the development and maturation of the oil palm fruit has been reported in the last decade, although still relatively fragmentary, namely (1) the de novo synthesis of fatty acids and their assembly in the pulp in *E. guineensis* (Montoya et al. 2013), (2) the transcriptional basis of oil and carotenoid biosynthesis during mesocarp development and maturation in palm fruits (Tranbarger et al. 2011), and (3) a comparison of fatty acid and sugar synthesis in oil palm and date palms (Bourgis et al. 2011). It is well established that K^+^ content in the fruit mesocarp increases progressively during the early stages of fruit formation up to 105 DAA, and then decreases during later development stages when lipid biosynthesis occurs (Mirande-Ney 2020). However, few data are available concerning K^+^ transport and its involvement in fruit formation and yield. Generally, in plants, several families of transporters and ion channels transport K^+^, among which *Shaker*-like K^+^ channels dominate the K^+^ conductance at the plasma membrane. *Shaker*-type K^+^ channels are voltage-dependent and are responsible for massive fluxes over long distances. These channels are involved in various biological processes such as the uptake of K^+^ from the soil, its secretion into xylem sap and the redistribution of this ion by phloem sap (Dreyer and Uozumi 2011). Voltage-dependent *Shaker*-type channels share a common structure composed of six transmembrane domains (S1–S6) featuring a selective pore region with the characteristic “GYGD” motif between S5 and S6 and a voltage sensor in S4. The transmembrane domains are followed by a large C-terminal region harboring a putative cyclic nucleotide-binding domain (CNBD) and a K_HA_ domain rich in acidic and hydrophobic amino acids involved in channel clustering (Ehrhardt et al. 1997; Pilot et al. 2003). Another motif within the C-terminus, not always present in all channels, is the ankyrin domain responsible for protein–protein interaction. *Shaker*-type channels are functional at the plasma membrane, when four monomers combine to form a central pore, either by homo-tetramerization, if the four subunits are identical, or by hetero-tetramerization by combining different subunits, which increases the functional diversity of the channel properties (Sharma et al. 2013; Véry et al. 2014). Plant *Shaker*-like channels are divided into five groups (I–V) according to their structural homologies and functional properties (Pilot et al. 2003; Gambale and Uozumi 2006). Specifically, these groups are distinguished by their current rectification, allowing directed K^+^ fluxes mediated by K_in_, K_out_, or K_weak_ channels. Groups I and II are inwardly rectifying channels (K_in_) that allow K⁺ uptake into the cytosol when the membrane is hyperpolarized, differing by the presence or absence of an ankyrin domain. Group III forms weakly rectifying channels (K_weak_), operating in two gating modes, that can mediate both K⁺ influx and efflux depending on the membrane potential and concentration gradient. Group IV consists of silent subunits that are not functional alone but form hetero-tetramers with K_in_ or K_weak_ channels to regulate their activity. Finally, Group V includes outwardly rectifying channels (K_out_) activated by membrane depolarization, which drive K⁺ efflux from the cytosol to the apoplast. Regarding transporters, in oil palm, three K^+^ transporters belonging to the K⁺ Transporter (KT), K⁺ Uptake Permease (KUP), and High-Affinity K⁺ transporter (HAK) families have been identified and characterized (Husri and Ong-Abdullah 2018, 2019), providing initial insights into K⁺ uptake mechanisms. In fact, the KT/KUP/HAK transporter family is known to be important for plants when subjected to low K^+^ concentrations (Nieves-Cordones et al. 2010). In oil palm, unlike the expression of *KUP8*, the expression of *KUP3* and *KUP11* does not appear to be sensitive to external K^+^ in roots and leaves (Husri and Ong-Abdullah 2018). Despite evidence that K⁺ content in the mesocarp peaks during early fruit development (up to 105 DAA) before declining when lipid biosynthesis starts (Mirande-Ney 2020), the molecular mechanisms underlying K⁺ transport, accumulation, and redistribution particularly via *Shaker*-type channels remain poorly understood. A complete dissection of these processes, both at the whole-plant level and during fruit development, is essential to improve K⁺ use efficiency to enhance oil palm crop performance. To address these questions, we analyzed the *Shaker*-like K⁺ channel family in oil palm and provide a first functional description of EgKT2-1, a member of the AKT2-like subfamily (Group III), which is highly expressed in fruits, whereas *EgKT2-2* does not show any detectable expression in fruits, as indicated by the absence of transcripts in real-time PCR experiments (S.E. and T.C., unpublished data). We report the expression profile of *EgKT2-1*, its cellular localization analyzed by in situ hybridization, and its functional characterization in *Xenope laevis* oocytes. Comparative analyses between two contrasted crosses concerning the production of Fresh Fruit Bunches (FFB) and crude palm oil (CPO) (C2 and C3) suggest that EgKT2-1 contributes to oil production, likely by enhancing K⁺ availability in mesocarp cells during early fruit development. Altogether, this study offers critical insights for improving K⁺ use efficiency ultimately aiming at reduction of fertilizer use and promote an eco-responsible management of oil palm groves.

## Materials and methods

### Plant materials

The experimental field was located at the Ologbo Estate of Presco Plc (Edo State, Nigeria, N 6.03652°, E 5.55609°, at an elevation of 20 m a.s.l). The trees were planted in 2011. The material from adult trees (flowers, fruits, leaves and leaf rachis) came from two crosses (C2 and C3) of the same origin (Deli x La Mé). C2 from DA 115 D AF x (LM 5 T × LM 10 T) and C3 from DA 115 D AF × LM 5 T AF; AF refers to self-pollinated trees. These crosses were selected for this study because, although they share the same origin, they exhibit different yields with the same K⁺ fertilizer application in Nigeria (Dassou et al. 2022a, b). The C3 progeny was recently reported to yield more oil and more bunches than the C2 progeny (Dassou et al. 2022a). Fruits were collected at three stages of development: early fruit development (30–60 DAA), mid-maturity (around 120 DAA), and maturity (around 160 DAA). Each organ was collected from a total of 27 palms. Mesocarp, leaves, and rachis tissues were placed in RNA stabilization reagent (RNAlater™, Qiagen) and stored at − 20 °C prior to RNA extraction. Approximately 100 mg of material was ground in liquid nitrogen and used for RNA extraction. The plants of Deli x La Mé origin were grown in the greenhouse for 5 months. The fresh tissues (young leaves, rachis, roots and meristem) were frozen in liquid nitrogen and stored at − 80 °C until RNA extraction.

### Isolation of cDNA encoding the EgKT2-1 protein

The reference genome sequence of oil palm (Jin et al. 2016) enabled in silico analysis for cloning of the selected *EgKT2-1* gene (LOC105036922). Specific primers (EgKT2-1-full-F 5’-ATGATGAATTGGTCCTTGAGCAAG-3 and EgKT2-1-full-R 5’-TCAAAGAAATTCTTCATTCTCAACTATGAAC-3) were designed based on the predicted sequences, and the corresponding cDNAs were isolated from leaf RNA.

### RNA extraction and real-time PCR analyses

The protocol proposed by Argout et al. (2008) was used to extract RNA from fruits. For the other tissues, total RNA was extracted using the Plant RNeasy extraction kit (Qiagen, Germany). After DNase I treatment (RQ1 RNase-free DNase, Promega, Madison, WI, USA; Cat. No. M610A), total RNA was eluted in 50 µl and quantified in an Agilent TapeStation 4200 System (Agilent Technologies, Santa Clara, CA, USA, https://www.agilent.com). Total RNA (500 ng) was used as the template to synthesize first-strand cDNA with SuperScript III reverse transcriptase (Invitrogen™, Carlsbad, CA, USA; Invitrogen Cat. No. 18080–093), according to the manufacturer’s instructions. Oligonucleotides were designed using Primer3Plus (https://primer3plus.com). All primers were designed in the 3’ region of each gene. The tubulin alpha-3 chain (TUA) (LOC105054774) gene (TUA5_F 5´-TGGAGAAGGGATGGAAGAAG-3´ and TUA5_R 5´-GGGAGAGGGAGAGAGAAACC-3´) was validated and used as housekeeping gene. The oligonucleotides used for *EgKT2-1* were EgKT2-1_F: 5’-ATACTCCCTTATGGCTCGCC-3’ and EgKT2-1_R: 5 ‘-CGAGAAGATTGCCACCGATG-3’. PCR reactions were performed on a LightCycler 480 II System (Roche Diagnostics, Mannheim, Germany) using SYBR Green I Master Mix (Roche Diagnostics, Mannheim, Germany; Cat. No. 04707516001) and gene-specific primers in 384-well plates according to the manufacturer’s specifications. Biological replicates consisted of individual oil palm trees. The numbers of biological replicates were as follows: leaves (*n* = 20), flowers (*n* = 3), rachis (*n* = 13), early fruit development (*n* = 19), mid-development fruits (*n* = 19), and mature fruits (*n* = 24). For comparisons between progenies, fruits were collected from individual palms. For progeny C2, 13 fruits at early fruit development, 15 mid-development fruits, and 11 mature fruits were analyzed, whereas for progeny C3, 6 fruits at early fruit development, 8 mid-development fruits, and 9 mature fruits were analyzed. For greenhouse-grown material, at least three individual palms were used as biological replicates for each tissue type: young leaves (*n* = 4), young rachis (*n* = 3), shoot meristem (*n* = 3), and roots (*n* = 3). In addition, each biological sample was analyzed in at least two technical replicates with the automated system (Biomek FXp).

Before RT-qPCR normalization, the expression variability of five candidate reference genes was evaluated across all tissues, genotypes, and both Elaeis species. Among these candidates, tubulin gene (TUA) exhibited the lowest variability (SD = 1.05) and was therefore selected as the reference gene for normalization. Relative expression levels were calculated using the 2−ΔCt method (Livak and Schmittgen 2001). Gene expression was normalized to the tubulin (TUA) reference gene, and ΔCt was calculated as ΔCt =Ct(target gene)−Ct(TUA). Expression data are presented as the mean 2−ΔCt values ± standard error of the mean (SEM). Differences among samples were assessed using the non-parametric Kruskal–Wallis test followed by Dunn’s multiple comparisons test with Bonferroni correction using XLSTAT (Addinsoft, Paris, France). The Bonferroni-adjusted significance threshold was determined according to the number of pairwise comparisons performed for each analysis.

### Localization of EgKT2-1 transcripts by mRNA in situ hybridization

*EgKT2-1*-specific primers EgKT2-1_F and EgKT2-1_R used for RT-qPCR were also used to synthesize RNA probes according to the protocol described by Alemanno et al. (2008). The specificity of the 105-bp in situ hybridization probe sequence was verified by BLAST searches against the oil palm genome available at NCBI, the new African oil palm reference genome derived from the La Mé population (Daval et al. 2026), and a private genome derived from the Deli genotype. Only the *EgKT2-1* sequence produced a significant match and indicated that cross-hybridization with *EgKT2-2* or other members of the Shaker K⁺ channel family is unlikely. Sense probe (used as control) and antisense probe were labeled with UTP-digoxigenin *during* transcription (T7 MAXIScript Kit, Ambion, Austin, TX, USA). Fruits, roots, and leaves were fixed overnight in 4% paraformaldehyde under RNase-free conditions, dehydrated through an ethanol series, and transferred to butanol for 2 weeks prior to paraffin embedding (Leuty 1969; Ferreira et al. 2017). Samples were then sectioned into 10 µm-thick cross sections using a microtome (Leica RM2125), and mounted onto silanized slides (Dako, Trappes, France). Hybridization was performed overnight at 45 °C. Slides were incubated with anti-DIG antibody combined with alkaline phosphatase (Roche Diagnostics, Mannheim, Germany; http://www.roche.com) diluted 1:500 for 1 h at 37 °C. Vector blue KITIII (Vector Laboratories, http://www.vectorlabs.com) was used to detect hybridization signals. In situ hybridization was performed independently on three individual palm trees (biological replicates). For each biological replicate, at least two independent tissue sections were analyzed. Signals were observed with a bright field microscope (Nikon Eclipse NI-E) equipped with 4x, 10× and 20× objectives. Images were captured using a DS-Ri2 camera (Nikon Instruments Inc., Tokyo, Japan), and scale bars were added using ImageJ (National Institutes of Health, Bethesda, MD, USA).

### Functional characterization of EgKT2-1

For functional characterization in *X. laevis* oocytes, in vitro transcriptions were performed using the mMESSAGE mMACHINE T7 Ultra Transcription Kit (Invitrogen™, Thermo Fisher Scientific, Waltham, MA, USA; Cat. No. AM1345) following the manufacturer’s instructions to obtain *EgKT2-1* cRNA. The *EgKT2-1* cRNA (6 ng in 12 nL) was microinjected into *X. laevis* oocytes using a microinjector (Nanoliter 2010, WPI, https://www.wpiinc.com). Control oocytes were injected with an equivalent volume of water (12 nL). Injected oocytes were stored at 20 °C in ND96 solution (mM: NaCl 96, KCl 2, MgCl_2_ 1, CaCl_2_ 1.8, HEPES 5, Na-pyruvate 2.5; gentamycin 50 µg ml^−1^, pH 7.4). Oocyte’s membrane currents were recorded 2–4 days postinjection, using a two-microelectrode voltage-clamp amplifier (GeneClamp 500B; Axon Instruments, Union City, CA, USA; http://www.moleculardevices.com), and a Digidata 1322A interface (Axon Instruments), with microelectrodes filled with 3 M KCl. Recordings were visualized and stored with pClampex 10 software (Molecular Devices, Sunnyvale, CA, USA).

During recordings, oocytes were maintained in perfusion solutions containing 100 (K100), 50 (K50), or 10 mM (K10) K^+^ gluconate, supplemented with 0, 50, or 90 mM Na⁺ gluconate, respectively, and containing: (mM) 1 CaCl_2_, 2 MgCl_2_, and 10 HEPES (pH 6.5 or 7.5) or 10 MES (pH 5.5). Channel selectivity was tested using (mM) KCl 100, NaCl 100, LiCl 100, and RbCl 100 solutions. Inhibition by Cs^+^ or Ba^2+^ was analyzed by adding 10 mM CsCl or BaCl_2_ to the K100 solution at pH 6.5. A voltage-clamp protocol was applied with 1.4 s voltage pulses from +70 mV to − 170 mV, in − 15 mV steps, followed by a 250 ms voltage step to − 40 mV at a holding potential of 0 mV. Current–voltage (I/V) curves were obtained by plotting steady-state currents measured at the end of voltage pulses against the corresponding membrane potentials. Data were analyzed using Clampfit 10 software (Axon Instruments).

## Results

### Phylogenetic relationships of EgKT2-1

**Fig. 1:**
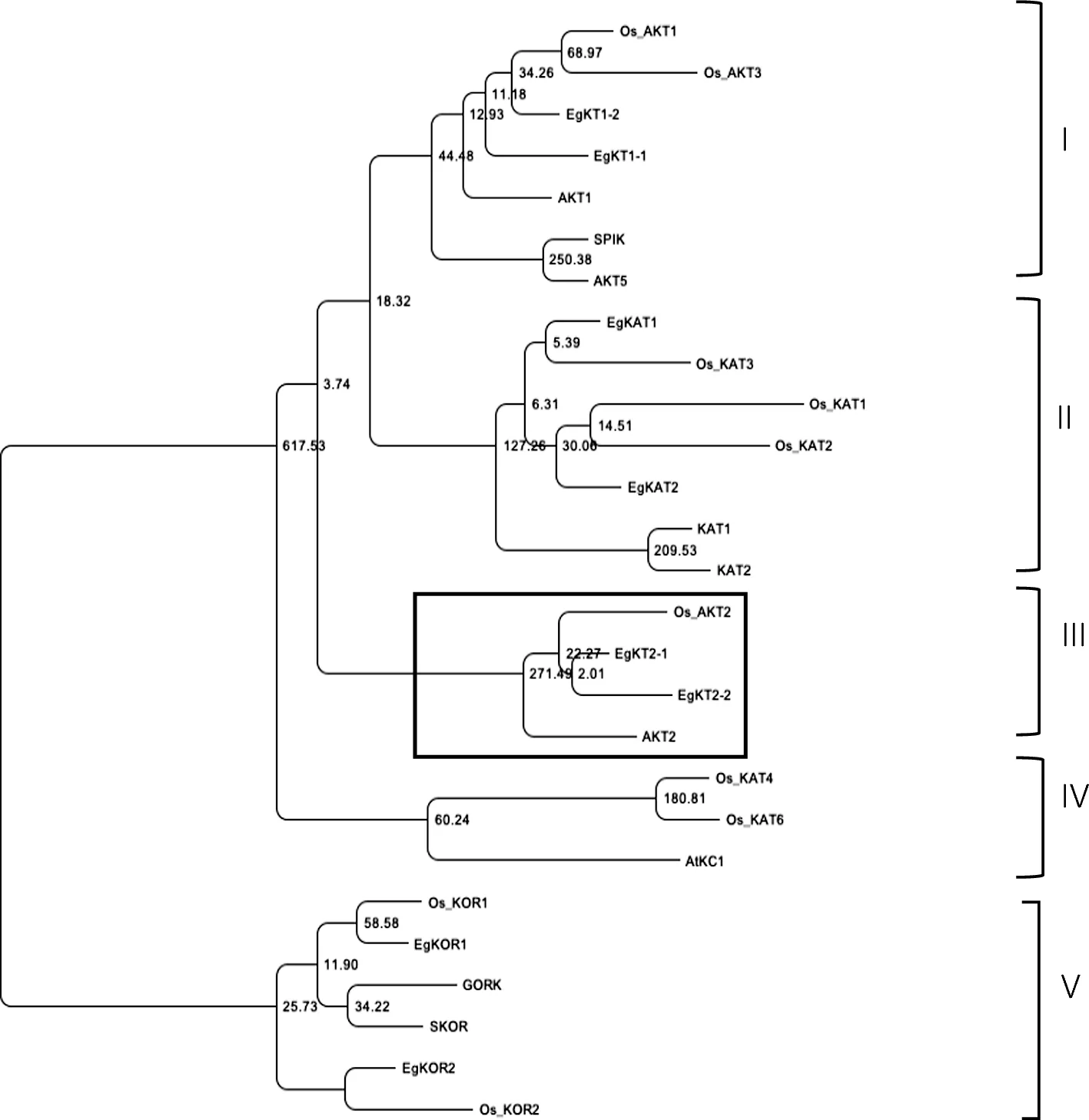
Phylogenetic relationships of *Shaker*-like channels in Arabidopsis, rice, and oil palm

Using the EguV3 reference genome (Jin et al. 2016), we identified eight sequences corresponding to genes encoding *Shaker*-type K^+^ channels in oil palm and constructed a phylogenetic tree to classify them. Phylogenetic analyses confirmed that the *Shaker*-like K^+^ channel family in oil palm includes eight members, consistent with the number described in date and coconut palms (Yang et al. 2021) while *Arabidopsis thaliana* (hereafter Arabidopsis) harbors nine (Sharma et al. 2013) and rice ten channels (Véry et al. 2014) (Fig. 1A). These oil palm channels were classified as follows: four members, EgKT1-1, EgKT1-2, EgKAT1, and EgKAT2, belong to Groups I and II of the inwardly rectifying (K_in_) channel subfamily, two subunits, EgKOR1 and EgKOR2, belong to Group V of the outwardly rectifying (K_out_) channel subfamily, and the remaining two members, EgKT2-1 and EgKT2-2, belong to Group III of the weakly rectifying (K_weak_) channel subfamily. Interestingly, oil palm, like other palm species, completely lacks the silent channel group (AtKC1-like) found in other model plant species. A notable feature in palms is the presence of two AKT2-like subfamily members (EgKT2-1 and EgKT2-2), whereas only a single member (AtKT2) has been previously described as weakly rectifying channel in the model species Arabidopsis (AtKT2) (black box in Fig. 1).

A phylogenetic tree was constructed using NGPhylogeny.fr software (Lemoine et al. 2019) (mode, selecting a combination of MAFFT, TrimAl, and PhyML in a Galaxy Workflow) and the InTreeGreat web interface (Dufayard et al. 2021) using the complete set of *Shaker* channel polypeptides of Arabidopsis, rice, and oil palm. The tree was manually pre-rooted with forester.jar application (https://www.phylosoft.org/archaeopteryx). *Shaker* channels are divided into five groups: Group I, the AKT1 subfamily, comprises inwardly rectifying channels with ankyrin domains. Group II, the KAT1 subfamily, harbors inwardly rectifying channels without the ankyrin domain. Group III, the AKT2 subfamily, is defined by channels with weakly rectifying K^+^ channels. Group IV, the AtKC1 subfamily, includes subunits that modulate other inwardly rectifying channels. Group V, the SKOR/GORK subfamily, gathers outwardly rectifying K^+^ channels. The Arabidopsis *Shaker*-like K^+^ channel family has nine members; the rice family ten members. In oil palm, the Shaker K^+^ channel family is composed of eight members. Note the presence of two oil palm members, EgKT2-1 and EgKT2-2, within Group III, in the so-called AKT2-like subfamily (black box), whereas only one member was found in Arabidopsis and in rice. Note the absence of Group IV in oil palm.

NCBI RefSeq gene symbols: EgKT1-1 (KT105047848), EgKT1-2 (LOC105047770), EgKAT1 (LOC105043816), EgKAT2 (LOC105040639), EgKT2-1 (LOC105036922), EgKT2-2 (LOC105051829), EgKOR1 (LOC105032653), EgKOR2 (LOC105051554); AKT1 (AT2G26650), SPIK (AT2G25600), AKT5 (AT4G32500), KAT1 (AT5G46240), KAT2 (AT4G18290), AKT2 (AT4G22200), AtKC1 (AT4G32650), GORK (AT5G37500), SKOR (AT3G02850); Os_AKT1 (LOC4326245), Os_AKT3 (LOC9271501), Os_KAT1 (LOC4328864), Os_KAT2 (LOC4325560), Os_KAT3 (LOC4325143), Os_AKT2 (LOC4338867), Os_KAT4 (LOC107276707), Os_KAT6 (LOC107276718), Os_KOR1 (LOC4340653), Os_KOR2 (LOC4335955).

In this study, we focused on the characterization of one of the two AKT2-like channels in oil palm, named EgKT2-1, due to its high expression during the early stages of fruit development (*Cf*. gene expression results). A 2511 bp full-length cDNA *EgKT2-1* (LOC105036922) was amplified using specific primers. The deduced EgKT2-1 polypeptide contains 836 amino acids and shares 73.86% and 68.71% identity with AtKT2 and OsKT2, respectively. The second EgKT2 channel (EgKT2-2) encodes a deduced polypeptide of 833 amino acids and shares 63.19% identity with OsAKT2 and only 56.9% identity with AKT2 (Arabidopsis). The two EgKT2 channels share 71.2% sequence identity. EgKT2-1 has the typical characteristics of a *Shaker* channel: six transmembrane segments with the K^+^ selective hallmark sequence ‘TXXTXGYGD’, followed by a large C-terminal domain containing a cyclic nucleotide-binding domain (CNBD), an ankyrin domain, and a K_HA_ domain. These structural features confirm its classification within the Shaker-like K⁺ channel family.

### Differential EgKT2-1 expression across roots, leaves, and fruits is associated with oil yield variation in oil palm crosses C2 and C3

**Fig. 2:**
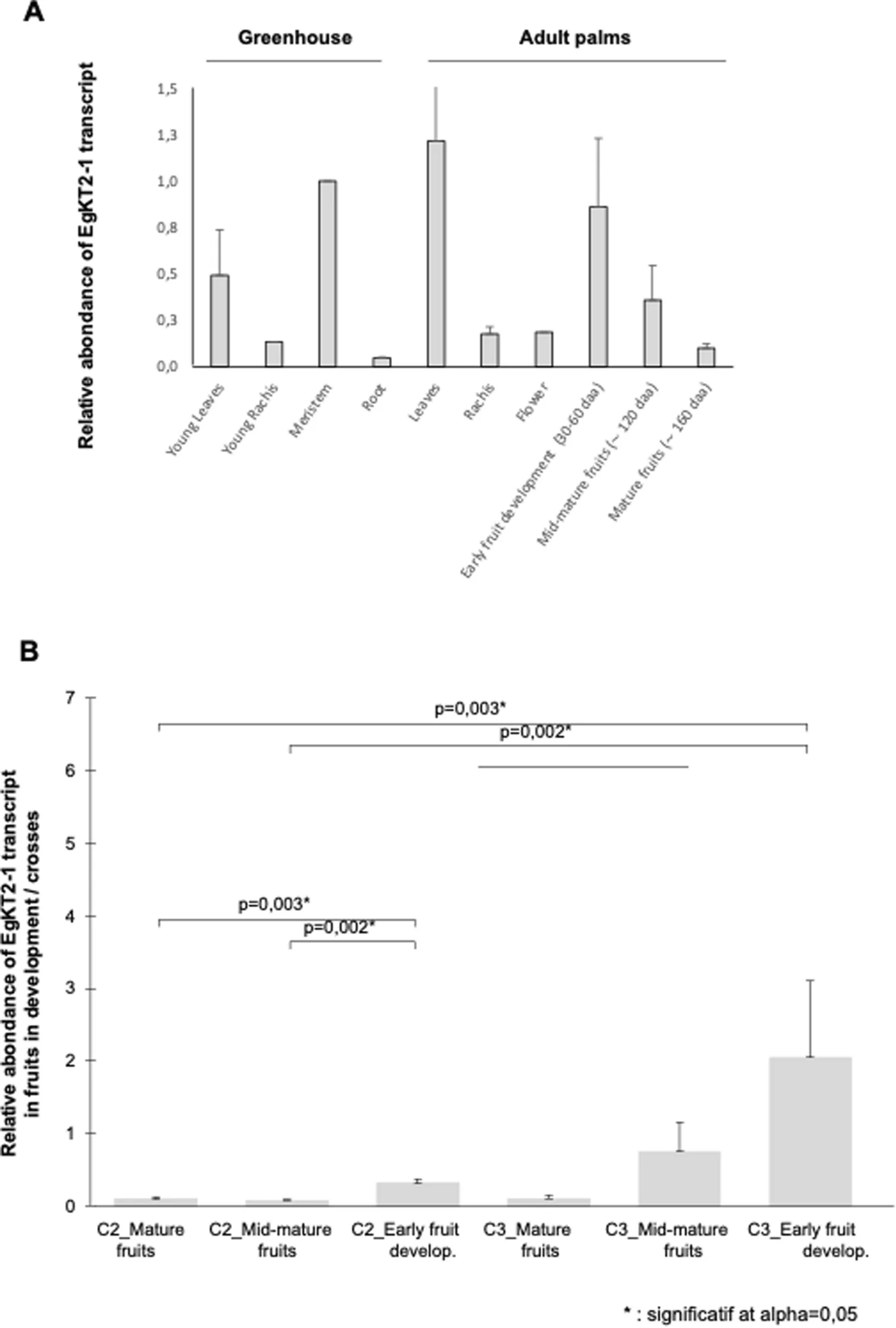
Relative *EgKT2-1* transcript abundance in oil palm tissues from greenhouse-grown seedlings and adult palms (**A**), and in fruits from adult palms of two crosses (**B**). Relative *EgKT2-1* transcript levels were determined by RT-qPCR using total RNA extracted from leaves, rachis, meristems, and roots of greenhouse-grown seedlings, and from leaves, rachis, flowers, and fruits at three developmental stages of adult palms (A). *EgKT2-1* was highly expressed in leaves, meristems, and fruits during early fruit development consistent with the reported increase in K⁺ accumulation during the early stages of fruit development. In contrast, *EgKT2-1* expression was low in flowers, roots, rachis, mid-development fruits (approximately 120 DAA), and mature fruits (160 DAA). Relative *EgKT2-1* transcript abundance was also analyzed in fruits from two Deli × La Mé crosses, C2 and C3 (B). *EgKT2-1* expression was higher in C3 fruits than in C2 fruits. Notably, this higher expression is associated with the greater oil and bunch yields previously reported for the C3 progeny compared with the C2 progeny under Nigerian growing conditions and identical K⁺ fertilizer application (Dassou et al. 2022a,b). For field-grown material, each biological replicate consisted of one individual oil palm. The numbers of biological replicates were as follows: leaves (*n* = 20), flowers (*n* = 3), rachis (*n* = 13), early-development fruits (*n* = 19), mid-development fruits (*n* = 19), and mature fruits (*n* = 24). Among the fruit samples, progeny C2 included 13 early-development fruits, 15 mid-development, and 11 mature fruits, whereas progeny C3 included 6 early-development fruits, 8 mid-development, and 9 mature fruits. For greenhouse-grown material, at least three individual palms were used as biological replicates for each tissue type: young leaves (*n* = 4), young rachis (*n* = 3), shoot meristem (*n* = 3), and roots (*n* = 3). Each biological sample was analyzed with at least two technical replicates. Expression data are presented as the mean 2−ΔCt values ± standard error of the mean (SEM). Differences in gene expression among samples were assessed using the non-parametric Kruskal–Wallis test, followed by pairwise comparisons using Dunn’s multiple comparisons test with Bonferroni correction (α = 0.0011 for panel A and α = 0.0033 for panel B)

Analyses of gene expression by RT-qPCR showed that *EgKT2-1* was expressed in all tested tissues. Notably, *EgKT2-1* was strongly expressed in leaves and meristems of young greenhouse plants. In adult palms, the highest expression levels were detected in leaves and fruits during early fruit development (30–60 DAA). In contrast, *EgKT2-1* showed low expression levels in flowers, roots, rachis, mid-ripening fruits (around 120 DAA), and mature fruits (160 DAA) (Fig. 2A).

Moreover, we analyzed *EgKT2-1* expression levels in the fruits from two crosses C2 and C3. Both crosses are hybrids derived from the same parental populations (maternal Deli population x paternal La Mé population) and were cultivated in Nigeria under identical K^+^ fertilizer conditions. However, the C3 progeny produced significantly more oil and bunches than the C2 progeny under the same growing conditions (Dassou et al. 2022a,b). Remarkably, the expression level of *EgKT2-1* in fruits from the C3 cross was almost fourfold higher than that observed in fruits from the C2 cross (Fig. 2B), a potential contribution of EgKT2-1 to K⁺ transport. This higher expression of *EgKT2-1* may imply a greater K^+^ influx into the mesocarp cells.

**Fig. 3:**
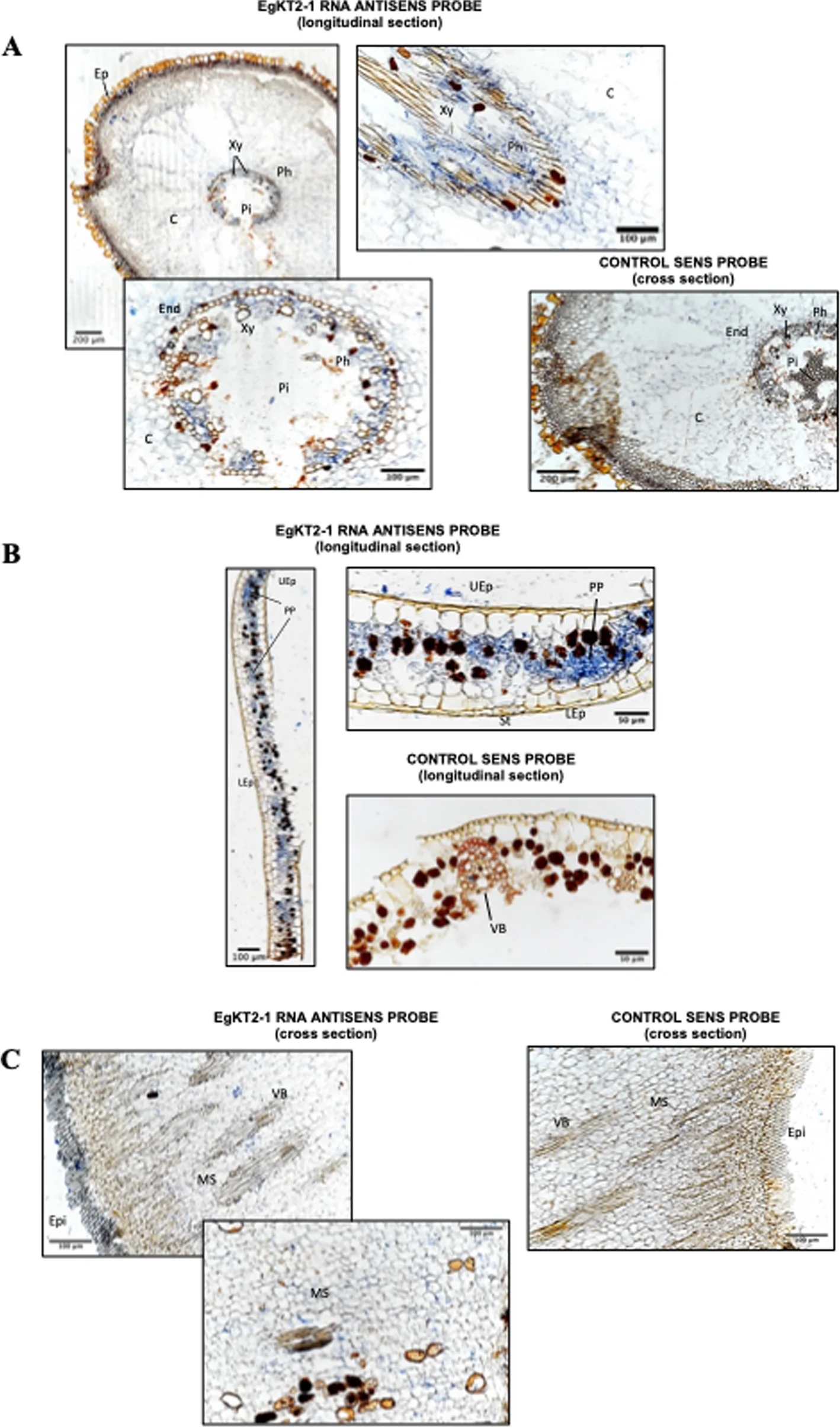
In situ localization of *EgKT2-1* transcripts in roots (**A**), young leaves (**B**), and fruits at early fruit development (60 DAA) (**C**). In situ hybridization was performed independently on three individual palm trees (biological replicates). For each biological replicate, at least two independent tissue sections were analyzed. Cross and longitudinal sections were hybridized with an *EgKT2-1* RNA antisense probe or RNA sense probe. Sections hybridized with the sense probe (negative control) showed no detectable signal. A positive blue signal was observed in roots, young leaves, and fruits at early fruit development with the *EgKT2-1* RNA antisense probe. In roots, the signals were detected in the phloem and cortex cells in both longitudinal and transversal sections (A). In young leaves, the blue signals were observed in cells of the palisade parenchyma of mesophyll (B). In fruits, the signals were detected in the mesocarp cells only in fruits at 60 DAA (C). C, cortex; End, endodermis; Epi, epicarp; LEp, lower epidermis; MS, mesocarp; Pe, pericycle; PP, palisade parenchyma; Pi, pith; Ph, phloem; St, stomata; Rh, rhizodermis; VB, vascular bundles; UEp, upper epidermis; Xy, xylem; DAA, days after anthesis

To further characterize the spatial expression pattern of *EgKT2-1*, we performed tissue localization studies using in situ hybridization (Fig. 3). No signal was detected with the sense probe used as a negative control in any tissue. In roots, transcripts were localized in the root cortex and phloem of young seedlings (Fig. 3A), as observed in both cross and longitudinal sections. Notably, strong labeling was also detected in the root pericycle, particularly in pericycle cells located adjacent to the xylem poles (Fig. 3A).

In leaves, hybridization signals were observed in palisade parenchyma cells (mesophyll) (Fig. 3B), whereas no labeling was detected in vascular bundles or stomata. In fruits, in situ hybridization was performed at three key developmental stages: early fruit development (60 DAA), mid-ripening (120 DAA), and maturity (160 DAA).

*EgKT2-1* transcripts were exclusively localized in mesocarp cells during early fruit development (60 DAA) (Fig. 3C), a developmental stage previously associated with high K⁺ accumulation.

### EgKT2-1 mediates inwardly rectifying K^+^ currents in *Xenopus laevis* oocytes

**Fig. 4:**
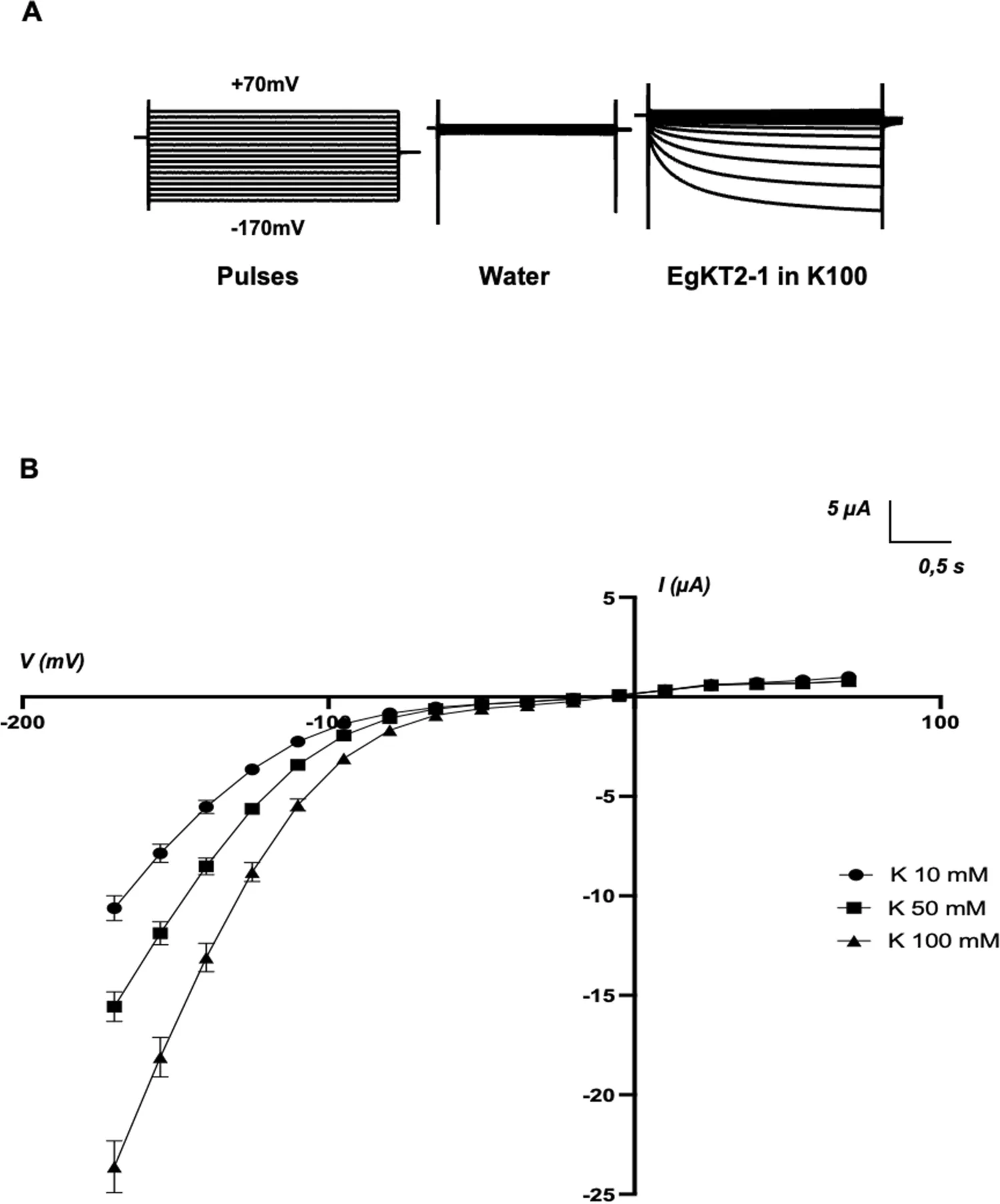
EgKT2-1 is an inwardly rectifying channel mediating inward potassium currents. The functional properties of EgKT2-1 channels were investigated in *Xenopus laevis* oocytes by voltage-clamp analyses. (**A**) Representative current traces in response to voltage pulses from +70 to − 170 mV for oocytes injected with water as control or with *EgKT2-1* cRNA (6 ng) in external bath solution of 100 mM K^+^ gluconate (K100), pH 6,5. (**B**) Current–voltage curves for mean currents mediated by *EgKT2-1* in different external K^+^ concentrations (K10, K50, and K100 mM), pH 6,5 (*n* = 15, ± SE)

The oil palm *Shake*r-like K^+^ channel EgKT2-1 was heterologously expressed in *X. laevis* oocytes and membrane currents were measured and compared to those of control oocytes (Fig. 4).

**Fig. 5:**
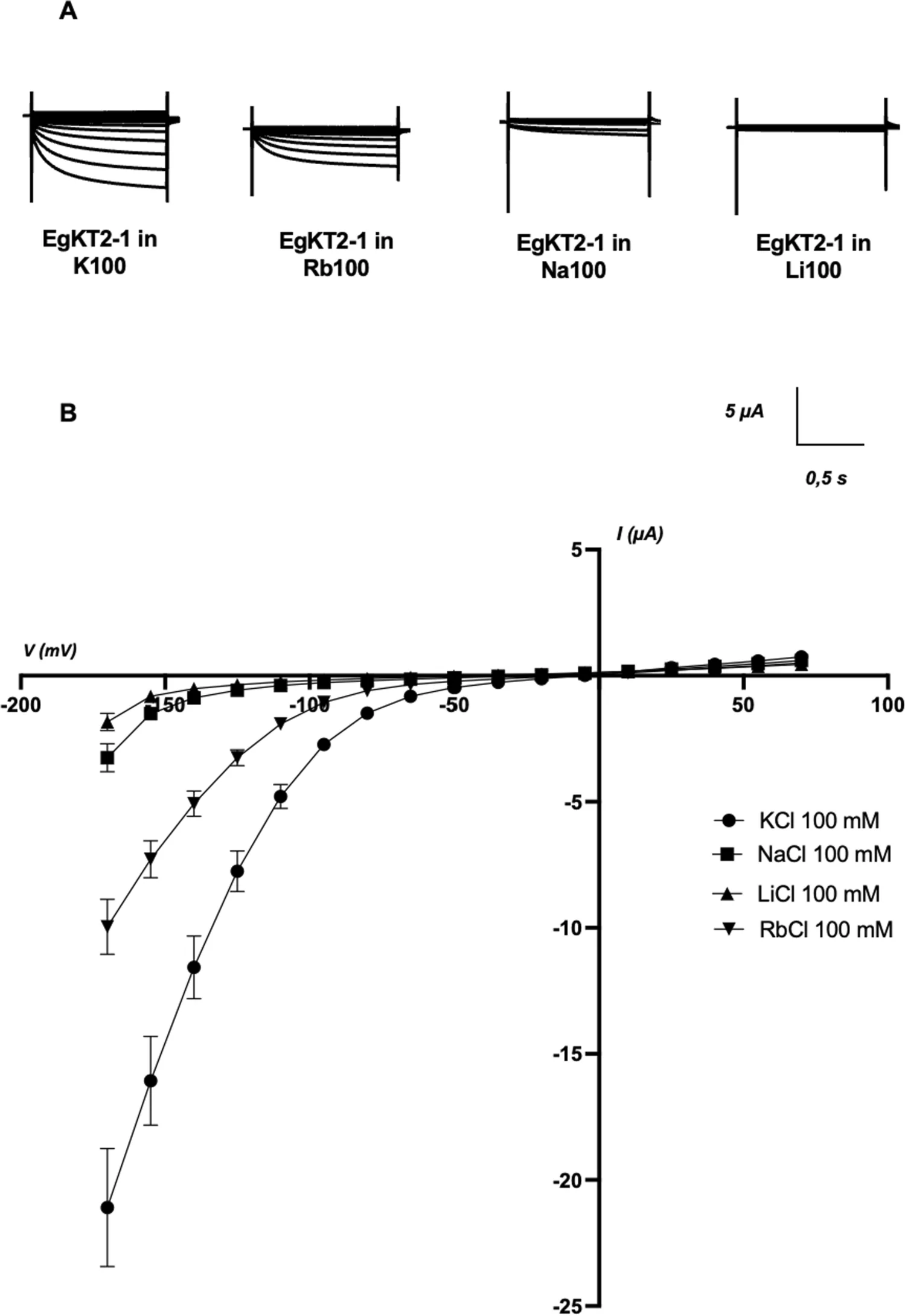
EgKT2-1 is selective for potassium. Permeability of EgKT2-1 for four monovalent cations was tested by electrophysiology in oocytes. (**A**) Representative current traces in response to voltage pulses from +70 to − 170 mV for oocytes expressing EgKT2-1 in 100 mM cation (K^+^, Rb^+^, Na^+^, and Li^+^) chloride salt, pH6.5. (**B**) Current–voltage curves for mean currents mediated by EgKT2-1 in 100 mM K^+^, Rb^+^, Na^+^, and Li^+^ bath solutions at pH 6,5 (*n* = 11, ±SE). Note that only Rb^+^ could replace partially K^+^

While water-injected control oocytes did not display any detectable currents, *EgKT2-1*-injected oocytes displayed voltage- and time-dependent inwardly rectifying currents upon membrane hyperpolarization in an external external K^+^ (100 mM) solution (Fig. 4A). These time-dependent activating currents showed sigmoidal activation kinetics, a hallmark of plant inwardly rectifying K^+^ channels. Varying external K^+^ concentrations caused a change in current amplitude with increased external K^+^ resulting in higher current amplitudes (Fig. 4B), confirming a K⁺-dependent conductance. Notably, the activation potential remained at around − 50 mV. Remarkably, large amplitudes of inward currents (up to − 25 µA) were observed, but outwardly directed (positive) currents remained negligible even at low external K^+^, indicating that any residual currents may result from endogenous oocyte currents or leak-like currents. Based on these findings that clearly revealed inwardly rectifying K^+^ currents, the oil palm channel EgKT2-1 belongs functionally to the subfamily of inward rectifiers and not to the weak rectifiers of the AKT2-type, as initially expected based on the phylogenetic relationship, which reflected sequence similarities. Further analyses of the selectivity of EgKT2-1 were performed to test the permeability of other monovalent cations. Ion currents were compared in the same oocytes expressing the oil palm channel in external solutions of 100 mM K^+^, sodium (Na^+^), lithium (Li^+^), and rubidium (Rb^+^) (all as XCl; Fig. 5).

**Fig. 6:**
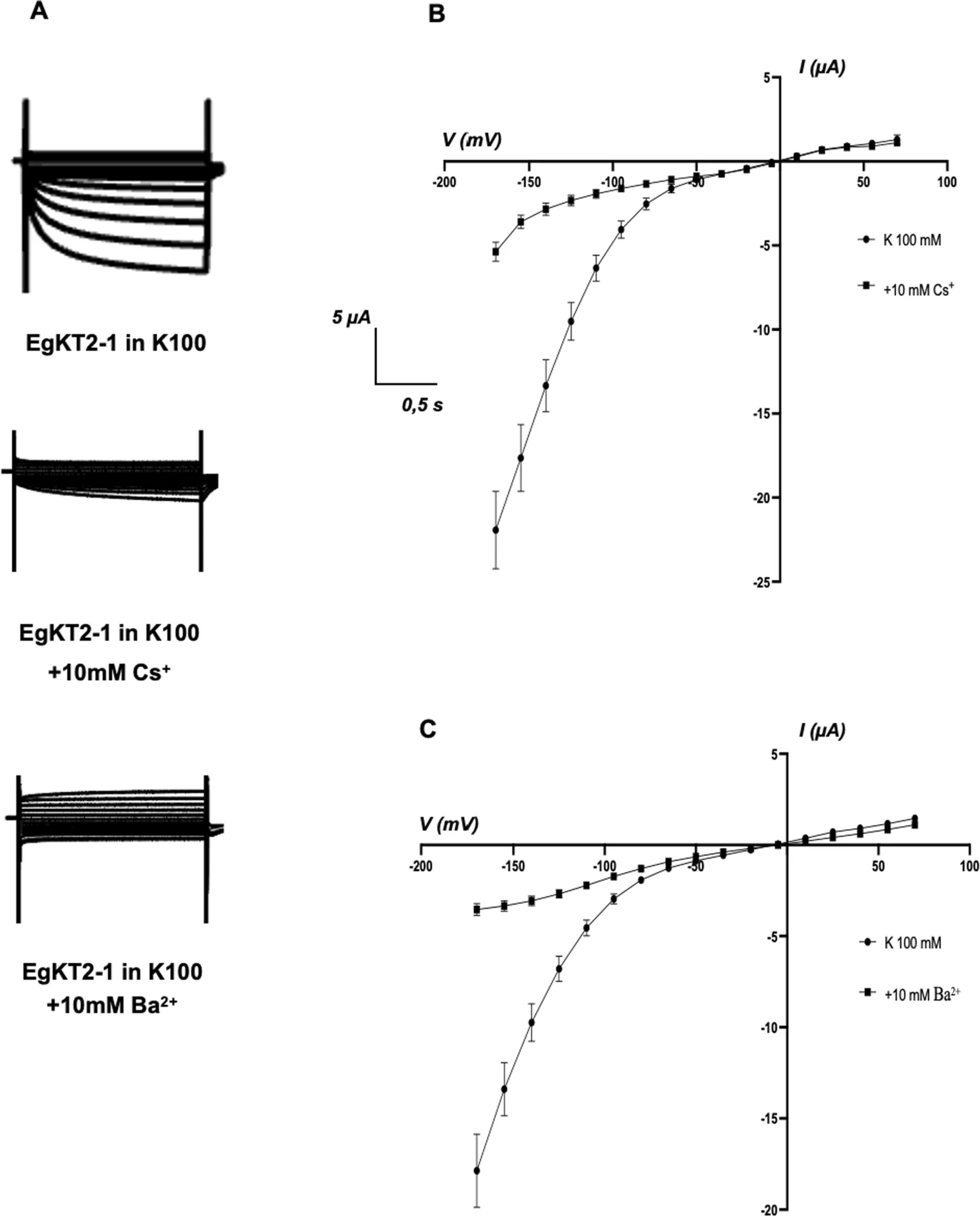
The oil palm channel EgKT2-1 is inhibited by external cesium (Cs^+^) and barium (Ba^2+^). (**A**) Representative current traces in response to voltage pulses from +70 to − 170 mV for oocytes expressing EgKT2-1 in an external 100 mM K^+^ solution as control or supplemented with 10 mM of Cs^+^ or Ba^2+^ at pH 6.5. Current–voltage curves are shown for EgKT2-1 currents recorded in 100 mM K^+^ with 10 mM Cs^+^ (**B**), and with 10 mM Ba^2+^ (**C**) at pH 6.5 (*n* = 8, ±SE)

Current traces in response to voltage pulses (Fig. 5A) demonstrated reduced amplitudes for all the other cations tested including Rb^+^ (K^+^ channels in plants are shown to be partially permeable to rubidium), compared with the K^+^ control condition. The mean currents plotted in current–voltage (IV) curves (Fig. 5B) showed reduced current intensities for Rb^+^ compared to that of K^+^. In parallel, (almost) no current was recorded for Na^+^ or Li^+^, suggesting that the remaining little currents were due to endogenous or leak-like currents. These results showed that EgKT2-1 is rather selective for K^+^ (K^+^>Rb^+^>>Li^+^,Na^+^)**.** It is also important to note that the activity of EgKT2-1 was not significantly sensitive to variations in external pH in the range of 5.5–7.5 in 100 mM external K^+^ (Supplementary Fig. S1). The pharmacological properties of the channel were then analyzed by external perfusion using the K^+^ channel blockers, Cs^+^ and Ba^2+^ (Fig. 6A). The results showed that EgKT2-1 currents were largely inhibited at all current-activating membrane potentials. At − 170 mV, the mean currents were reduced by 75% (10 mM CsCl, *n* = 8; Fig. 6B) and by 80% (10 mM BaCl_2_, *n* = 8; Fig. 6C). It should be noted that the inhibitory effect of these blockers was reversible upon return to a 100 mM K^+^ solution.

## Discussion

### EgKT2-1 behaves as an inward K^+^ channel

*EgKT2-1* was phylogenetically classified as an AKT2-like *Shaker* channel (Group III) based on its sequence similarity to other members of this subfamily (Fig. 1). AKT2-type channels are generally characterized by distinctive electrophysiological properties, including weakly rectifying currents, although exceptions have been reported in several species (Huang et al. 2021; Boscari et al. 2009). Unlike the model plant species Arabidopsis and rice, in which only one AKT2-type channel has been identified, the oil palm genome contains two members of this subfamily. Functional characterization of one of these channels, *EgKT2-*1, in *Xenopus laevis* oocytes revealed electrophysiological properties that differ from those of the canonical weakly rectifying AKT2 channel. EgKT2-1 mediated large inwardly rectifying K⁺ currents (Fig. 4A), consistent with a potential role in facilitating cellular K⁺ uptake. A similar atypical AKT2-type channel was recently described in rice (OsAKT2), which also behaves predominantly as an inward rectifier (Huang et al. 2021). Site-directed mutagenesis studies demonstrated that a lysine residue in the S4 domain (K191), replacing the conserved arginine found in Arabidopsis AKT2, is a major determinant of this rectification behavior. Concerning the oil palm channel EgKT2-1, amino acid sequence alignments between AtAKT2, OsAKT2, and EgKT2-1 revealed the presence of a single lysine residue (K195) in the S4 domain, corresponding to the K191 residue in OsAKT2 whereas AtAKT2 contains two consecutive arginine residues (R194 and R195) (Supplementary Fig. S2). This similarity between EgKT2-1 and OsAKT2 is consistent with the inwardly rectifying behavior observed for EgKT2-1. Taken together, these observations suggest that weak rectification is not a universal feature of AKT2-type channels but depends on key amino acid residues within the voltage-sensing domain. The presence of two AKT2 homologs in oil palm further raises the possibility that these channels fulfill distinct physiological functions, although this hypothesis remains to be experimentally tested.

Unlike several AKT2 orthologs from Arabidopsis, maize, grapevine, rice, and barley, whose activity is inhibited by external acidification (Philippar et al. 1999; Boscari et al. 2009; Nieves-Cordones et al. 2019; Huang et al. 2021), EgKT2-1 activity remained unaffected by changes in external pH (Supplementary Figure S1). This distinct pH response is intriguing because previous work on Arabidopsis AKT2 identified two residues located in the outer mouth of the K^+^-conducting pore, His-228 and Ser-271, as key determinants of proton sensitivity (Geiger et al. 2002). Sequence comparison showed that both residues are conserved in EgKT2-1, including the His residue immediately downstream of the selectivity filter motif (GYGD) and the Ser residue in the outer pore region. Therefore, the lack of pH sensitivity in *EgKT2-1* cannot be explained by the absence of these previously identified proton-sensitive residues. Instead, it suggests that pH regulation depends not only on the conservation of individual amino acids but also on additional structural determinants, such as the local environment surrounding these residues, neighboring amino acid interactions, pore conformational dynamics, or the efficiency of coupling between protonation events and channel gating. Although the overall pore region is highly conserved, subtle differences in structural context may alter the functional response to intracellular pH. The molecular basis of the pH insensitivity of *EgKT2-1* therefore remains unresolved and will require further structural and functional analyses.

Another distinctive feature of the oil palm *Shaker* family is the absence of an AtKC1-like silent channel subunit, a characteristic shared with coconut and date palm (Yang et al. 2021). However, variation in the composition of the *Shaker* family is not restricted to palms, as centipedegrass also lacks both Group III and Group IV members (Hao et al. 2025). These observations indicate that the composition of the Shaker channel family has diversified among plant species, potentially reflecting species-specific physiological requirements.

### Potential role of EgKT2-1 in K^+^ transport during oil palm fruit development

The expression analysis showed that *EgKT2-1* is expressed in both source and sink organs, with higher transcript abundance in meristematic tissues, leaves, and fruits at the early fruit development stage. This broad expression pattern differs from several AKT2 orthologs described in other species, which are often enriched in vascular tissues. For example, *AtAKT2* is mainly expressed in phloem tissues throughout aerial organs (Lacombe et al. 2000), whereas the rice *OsAKT2* and the grapevine *Vvk3.1* are predominantly associated with vascular tissues, and are thus involved in K⁺ redistribution during plant development (Nieves-Cordones et al. 2019; Huang et al. 2021).

In young roots, in situ hybridization revealed *EgKT2-1* expression in cortical and phloem tissues. Together with the inward K⁺ currents measured in *X.laevis* oocytes, this localization is consistent with a potential role of EgKT2-1 in K⁺ uptake and radial transport toward the stele. However, direct physiological evidence for this function in oil palm remains to be established.

Similar to *HvAKT2* in barley (Boscari et al. 2009), *EgKT2-1* transcripts were detected in mesophyll cells, specifically in the palisade parenchyma, whereas no expression was observed in stomata or vascular tissues. Because potassium contributes to mesophyll function and photosynthetic activity through its effects on enzyme activation and CO₂ conductance (Hu et al. 2022), this localization suggests that EgKT2-1 may contribute to K⁺ uptake in photosynthetically active tissues. Whether this activity directly affects photosynthetic performance in oil palm requires further investigation.

In developing fruits, *EgKT2-1* expression was strongest during early mesocarp development, with transcripts detected specifically in mesocarp cells at 60 DAA. This expression pattern coincides with the increase in fruit K⁺ content occurring before the major phase of lipid accumulation (around 120 DAA), suggesting that EgKT2-1 may contribute to K⁺ supply during early fruit development. The strict inward rectification of EgKT2-1 may be advantageous at this stage, as it favors K⁺ influx at negative membrane potentials while limiting K⁺ efflux under more depolarized conditions. This property could therefore facilitate K⁺ accumulation in developing mesocarp cells during a period of active fruit growth and increasing metabolic demand.

Potassium availability influences several processes involved in plant carbon metabolism, including pyruvate kinase and pyruvate dehydrogenase activities, which are linked to the production of precursors required for fatty acid synthesis (Cui and Tcherkez 2021). Consistently, previous studies in oil palm showed that low K⁺ availability affects pyruvate metabolism and mesocarp metabolism, although oil composition in mature fruits was not modified (Cui et al. 2019; Mirande-Ney et al. 2019). These observations support the hypothesis that K⁺ availability influences metabolic processes during early fruit development, but they do not establish a direct link between EgKT2-1 activity and oil biosynthesis.

Commercial oil palm varieties are generally tenera hybrids derived from crosses between heterotic group A (mainly of Deli origin, dura palms) and group B (mainly of African origin such as La Mé, pisifera palms). Two crosses, C2 and C3, derived from the same parental origins (Deli × La Mé), were used in this study under the environmental conditions prevailing in Nigeria and with identical K^+^ fertilizer applications. The comparison between these two genetically related progenies therefore provides an opportunity to explore a possible relationship between *EgKT2-1* expression and K⁺ allocation. The C3 progeny was previously reported to produce higher bunch and oil yields and to accumulate more K⁺ in aerial organs (Dassou et al. 2022a, b; Dassou 2023). The higher *EgKT2-1* expression observed in C3 is therefore consistent with a potential contribution of this channel to K⁺ distribution during fruit development.

Despite the clear correlation between EgKT2-1 expression and oil yield observed in this study, the association should be interpreted with caution, as it was inferred from comparisons between only two oil palm progenies. Although these progenies share the same parental origins, differences in genetic background other than EgKT2-1 expression may also contribute to the observed phenotypic variation. Therefore, the correlations reported here should not be interpreted as evidence of a direct causal role of *EgKT2-1* in oil accumulation. Likewise, even though the heterologous expression of EgKT2-1 in *X. laevis* oocytes demonstrates clearly that the protein functions as an inward K⁺ channel thus contributing to K⁺ transport, its impact on oil biosynthesis *in planta* is deduced by correlation. Moreover, the absence of *EgKT2-2* expression in developing fruits suggests that this channel may have a limited contribution to K⁺ transport during mesocarp development, although this hypothesis remains to be experimentally tested.

Taken together, our results show that *EgKT2-1* encodes a functional inwardly rectifying K⁺ channel and is expressed in tissues potentially involved in K⁺ acquisition, redistribution, and utilization during oil palm development. Its expression pattern, together with the higher transcript abundance observed in the higher-yielding C3 progeny, supports the hypothesis that EgKT2-1 may contribute to K⁺ allocation during early fruit development. Because K⁺ is required for carbon metabolism and the enzymatic processes associated with fatty acid synthesis, EgKT2-1 may indirectly contribute to establishing metabolic conditions favorable for oil accumulation. However, the present study establishes an association rather than a causal relationship. The observed association between EgKT2-1 expression and oil yield therefore suggests that EgKT2-1 could be considered a potential molecular marker candidate for oil production traits; however, the current data are insufficient to establish its reliability for breeding applications. Validation in larger, independent oil palm populations representing diverse genetic backgrounds and environmental conditions will be necessary to determine whether this association is robust and reproducible. Furthermore, functional studies, particularly genetic approaches, will be required to determine whether variation in EgKT2-1 expression or activity directly contributes to differences in oil yield and whether EgKT2-1 could ultimately be developed as a molecular marker for oil palm breeding programs.

## Conclusion

In conclusion, the functional characterization of EgKT2-1 demonstrates that it encodes a functional inwardly rectifying *Shaker*-type K⁺ channel in oil palm, as shown by its heterologous expression in *Xenopus laevis* oocytes. Its tissue-specific expression pattern, together with its electrophysiological properties, suggests that EgKT2-1 may fulfill distinct physiological functions depending on the organ. In roots, its localization in cortical and phloem tissues is consistent with a role in K⁺ uptake and radial transport, whereas its expression in palisade mesophyll cells suggests a potential contribution to K⁺ uptake or redistribution in photosynthetically active tissues. In developing fruits, the preferential expression of EgKT2-1 during the early stages of mesocarp development supports a potential role in K⁺ accumulation during fruit development.

The higher expression of EgKT2-1 in the progeny exhibiting greater K⁺ allocation to aerial organs and higher oil yield supports the hypothesis that this channel may contribute to K⁺ distribution within the plant. Given the central role of K⁺ in phloem transport, osmotic regulation, enzyme activation, and carbon metabolism, EgKT2-1-mediated K⁺ transport may contribute to physiological conditions favorable for fruit development and subsequent oil accumulation. However, the present study establishes a correlation rather than a causal relationship, and the proposed mechanistic link between EgKT2-1-mediated K⁺ transport and oil biosynthesis remains to be further validated.

Overall, this work provides new insights into the functional properties and tissue-specific expression of an AKT2-like *Shaker* channel in oil palm and identifies EgKT2-1 as a promising candidate involved in K⁺ transport and redistribution. Future studies combining genetic, physiological, and metabolic approaches, including transgenic approaches and CRISPR-based strategies to modify EgKT2-1 expression, will be needed to determine its precise biological role in K⁺ use efficiency and fruit development. In parallel, association analyses in larger and genetically diverse oil palm breeding populations will be necessary to determine whether EgKT2-1 expression or genetic variation is consistently associated with K⁺ use efficiency and oil yield, and whether could therefore be evaluated as a potential marker for improving potassium use efficiency in oil palm breeding programs.

## Supplementary Information

Below is the link to the electronic supplementary material.

Supplementary file1 (PDF 178 KB)

Supplementary file2 (PDF 121 KB)

## Data Availability

All data supporting the findings of this study are available within the paper and its Supplementary Information.
