# Supplementary material for: EgKT2-1, an inwardly rectifying Shaker-type potassium channel in oil palm that correlates with oil yield
Source: Plant Cell Rep. 2026 Sep 30;45(10):315. doi: 10.1007/s00299-026-03975-7 (PMC13627232; doi:10.1007/s00299-026-03975-7)
Supplement: Supplementary file 1 [file 299_2026_3975_MOESM1_ESM.pdf]

Figure S1

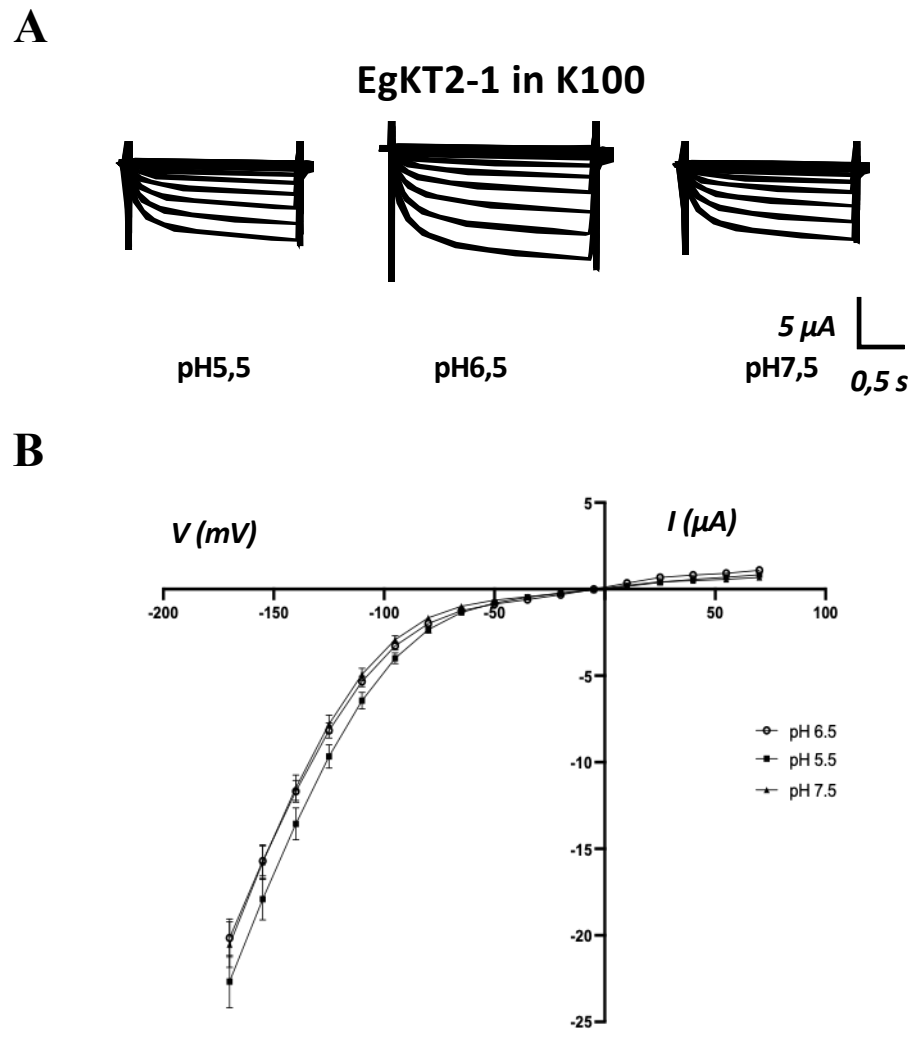

Figure S1: EgKT2-1 is not sensitive to variations in external pH.
The effect of external pH on EgKT2-1 was tested in a K100 bath solution with three different pH values, pH 6.5 as control, pH 5.5 and pH 7.5, respectively. (A) Representative current traces in response to voltage-pulses from +70 to -170 mV for oocytes expressing EgKT2-1 in external 100 mM K^+ bath solution at three different pH values (6.5, 5.5 and 7.5). (**B**) Current-voltage curves are shown for EgKT2-1 currents in 100 mM K^+ at pH 6.5, 5.5 and 7.5 respectively (n=15, ±SE).

Figure S2

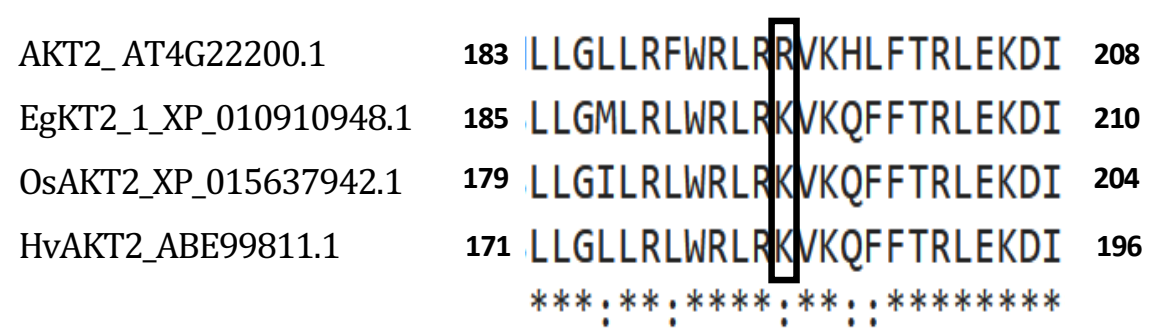

Figure S2: Amino acid sequence alignment on the S4 segment in AKT2, EgKT2-1, OsAKT2 and HvAKT2. Amino acid sequence alignment between four Shaker channels of the KT2-like subfamily (group III) on the S4 segment, focused on residues involved in rectification control. Arabidopsis, AKT2 (AT4G222200) ; oil palm, EgKT2-1 (LOC105036922), rice, OsAKT2 (LOC4338867) and barley, HvAKT2 (XP_044975261). Amino acid sequence alignment between AtKT2, EgKT2-1, OsKT2 and HvAKT2 revealed a single K195 residue similar to the K191 residue in OsKT2 and K183 in HVAKT2 versus two continuous RR residues (R194 and R195) in Arabidopsis. Only residue K191 (for OsAKT2), K195 (for EgKT2-1) or K183 (for HvAKT2) attributes control of rectification to OsAKT2, EgKT2-1 and HVAKT2 respectively and maintains these channels as inward rectifier channels only. In contrast Arabidopsis has two gating modes, one mediating K^+ (Kin) influx into the cell and the other activated by a time-dependent voltage mediate both K^+ efflux and influx.
