# Supplementary material for: EgKT2-1, an inwardly rectifying Shaker-type potassium channel in oil palm that correlates with oil yield
Source: Plant Cell Rep. 2026 Sep 30;45(10):315. doi: 10.1007/s00299-026-03975-7 (PMC13627232; doi:10.1007/s00299-026-03975-7)
Supplement: Supplementary file 2 [file 299_2026_3975_MOESM2_ESM.pdf]

Supplementary Table 1. Pairwise statistical comparisons of EgKT2-1 expression among oil palm tissues. P-values were obtained from Dunn's multiple comparisons test following Kruskal–Wallis analysis with Bonferroni correction. The Bonferroni-adjusted significance threshold was α = 0.0011. Significant comparisons are indicated in red.

	Flower	Leaves	Mature fruits (~ 160 daa)	Meristem	Mid-mature fruits (~ 120 daa)	Rachis	Root	Young Leaves	Young Rachis	Young fruits (30-60 daa)
Flower	1	0,893	0,366	0,516	0,507	0,726	0,334	0,953	0,798	0,793
Leaves	0,893	1	0,000	0,446	0,011	0,159	0,043	0,895	0,626	0,683
Mature fruits (~ 160 daa)	0,366	0,000	1	0,071	0,428	0,104	0,753	0,067	0,582	0,000
Meristem	0,516	0,446	0,071	1	0,119	0,216	0,078	0,445	0,365	0,526
Mid-mature fruits (~ 120 daa)	0,507	0,011	0,428	0,119	1	0,379	0,482	0,175	0,756	0,003
Rachis	0,726	0,159	0,104	0,216	0,379	1	0,240	0,453	0,998	0,079
Root	0,334	0,043	0,753	0,078	0,482	0,240	1	0,122	0,513	0,026
Young Leaves	0,953	0,895	0,067	0,445	0,175	0,453	0,122	1	0,703	0,712
Young Rachis	0,798	0,626	0,582	0,365	0,756	0,998	0,513	0,703	1	0,539
Young fruits (30-60 daa)	0,793	0,683	0,000	0,526	0,003	0,079	0,026	0,712	0,539	1
